# Supplementary material for: Patterns of Intron Gain and Loss in Fungi
Source: PLoS Biol. 2004 Nov 30;2(12):e422. doi: 10.1371/journal.pbio.0020422 (PMC532390; doi:10.1371/journal.pbio.0020422)
Supplement: Table S1 — Also available at http://genes.mit.edu/NielsenEtAl/. (4.3 MB ZIP). [file pbio.0020422.st001.zip › NielsenEtAl/html/1262.html]

AN4736.1.NCU01961.1.MG02980.1.FG08889.1


```
 CLUSTAL W (1.82) Multiple Sequence Alignments - Introns Inserted


Sequence 1: NCU01961.1	654 aa
Sequence 2: MG02980.1	626 aa
Sequence 3: AN4736.1	554 aa
Sequence 4: FG08889.1	609 aa
Alignment Length: 672 aa
Number Identitical Residues: 196 aa
Alignment Score (without introns) 10810


MG02980.1 	------------------------------~-MFDILEADIVVIQE~AKIQRKDLQDDMV
NCU01961.1	--MAGEPHVSGGIKFPIQKPYLAGCSNTVK~AMFDTLDADIVVIQE~TKIQRKDLRDDMV
FG08889.1 	MAFRLTTWNGEFIHFFRGPHTNTFCSQRHQ0SMFDILEADIVVMQE~TKIQQKDLLDEMV
AN4736.1  	------------------------------~------------MER2SVINLR-------
          	                                           ::. : *: :       

MG02980.1 	LVPGWDVYFSLPKFKKG~YSGVAIYTRSSKCSPIRAEEGITGILCPPNSTTKFRELSPDQ
NCU01961.1	LVPGWDVYFNLPTYKKG~YSGVAIYTRSSKCCPIRAEEGITGVLCPPNSSTKFRDLPADQ
FG08889.1 	LVPGWDVFFSLPKHKKG1YSGVAIYTRNSTCAPIRAEEGITGILTPPKSTTRYRDLPEDQ
AN4736.1  	-------------FAIG1YSGVAIYTRNATCAPIRAEEGLTGTLCPPNSLVSFRDLPEDQ
          	             .  * *********.:.*.*******:** * **:* . :*:*. **

MG02980.1 	QIGGYPRPDQLSD-IIDEATLDSEGRCMILEFPAFVLIGVYSPATRDETRTDFRQAFHKA
NCU01961.1	QIGGYPTPGQLSS-DIDEAQLDSEGRCVILEFPAFVLIGVYSPATRDETRIEFRQAFIDA
FG08889.1 	QIGGYPRPGQLDG-IIDEATLDSEGRCVILEFPGFVLLGVYSPANRDETRDDFRLGFLQA
AN4736.1  	QIGGYPTIEQLSKLKLDAETLDSEGRCVILEFPAFVLIGLYCPANRDESRDAFRQNFLDL
          	******   **.   :*   *******:*****.***:*:*.**.***:*  **  * . 

MG02980.1 	MDARVRNLVAMGKQVVLTGDLNIIRNELDTAGILERLRKEEMTIDEFFSTPSRRFLNHMV
NCU01961.1	MDARVRNLVSLGKEVFLCGDLNIIRNEMDTAGLPEQLKKEAMTLDQFISTPSRRFLNQIV
FG08889.1 	LDVRIRNLVAAGKQVVLTGDLNIVRSEVDSTNVSETLRKGNFDLNEWMNIPARRIFNQLI
AN4736.1  	MDARVRNLVALGKRVFVTGDINISRGEIDAAHAAENIKKGVTTEDDFVSAPARRLFNQLL
          	:*.*:****: **.*.: **:** *.*:*::   * ::*     :::.. *:**::*:::

MG02980.1 	FGGTVVGGRDEGREEPVMYDLGREFHPDRIGMYTCWETRKNARPGNFGSRIDYVLCSAGM
NCU01961.1	FGGRVIGEPDEGREKPVLWDLCREFHPKRPGMYTCWETRKNARPGNFGSRIDYVLCSSGI
FG08889.1 	FEGSVLGERDQGRKAPVLWDLCRCFHPERAGMNTCWDTKRNTRPANNGSRIDYVLCSDGI
AN4736.1  	IDGKVVGDRDEGREQPVLFDICRSFHPKRKGMYTCWEQRINARPGNYGSRIDYVLCSLDM
          	: * *:*  *:**: **::*: * ***.* ** ***: : *:**.* ********** .:

MG02980.1 	KDWFIDANIQEGLL~GSDHCPVYATLSDNVRTADGKSVPLVDIMNPKGMFVNGKRVRDWS
NCU01961.1	KDWFVDANIQEGLL~GSDHCPVYATISDSVR-RDGVIIPITDIMNPEGMFKNGQRQREWS
FG08889.1 	KSWFNFSNIQEGLM~GSDHCPVFAIFSDKVIVRGEE-RALPEMMNPPGMFHGERRLRDWN
AN4736.1  	KDWFFDSNIQEGLM0GSDHCPVYAVFKDLIPLNDGQ-SHILDIMNPPGVFKNGERQQNYT
          	*.**  :******: *******:* :.* :   .     : ::*** *:* . .* :::.

MG02980.1 	AKDLLPLSAKLIPDFDRRRSIKDMFFKKPKPTSS-RDVPSEIREETPSETAPPSPVTKSP
NCU01961.1	QKDCLPTSARLIPEFDRRQSIKDMFVKKKTPSNTPKSATPAADSQDISDSPNTSSITVNA
FG08889.1 	AKDLLPLSAKLIPEFDRRRSIRDMFTKKAASAAPPQEPANPGAATKSSMNGDSNSEGIKE
AN4736.1  	AKFLLPLSGRLIPEFDRRRSIKDMFMRKPSQPSPKTSSPQNLTACLSNEESSMTARTATN
          	 *  ** *.:***:****:**:*** :*   . .  . .        .     ..   . 

MG02980.1 	NTKEDDQSAKQDGRPSSQTSTSSSTVILPQTNPSIQKRPSKSSASNSRPQKKTKVST-SN
NCU01961.1	TTVLQGTASSASSPRQWLSQSTATDPALSQTSP-VPKRRAEQPAPYTRPPKKAKAAL-AK
FG08889.1 	--KSGGASGPETSAISIPKLGETLGSTKQSVSPLNAKRPGSVTDTTSRPLKKTKSLVGAK
AN4736.1  	TPKPSDADAPASVSNDTLQKGT------------VRKRPVGTEVP---PVKRSKSAS-TQ
          	..   .  .      .                    **      .   * *::*    ::

MG02980.1 	TAKNSVDRAPGQKSLTGFFKPKNPIST--PATDGERTSIR-TTPSPTKCSN-ASTPKIPG
NCU01961.1	QPSSKTGPGPSQSTLMGFIKAKTPTPT--VNPDERHGPINGNNHSPVTAAE-TALRNEP-
FG08889.1 	DLKSKVAPG--QRTLQGFFKPKAPAAQDGKSERVAASSTPSPTKKPTVATRGAASPQQPN
AN4736.1  	TGAPTIG----QSTLKGFFKPKCTLEP--KDNMLSKDEGQGPAEFPFISSQ-QDVTEQT-
          	    .      * :* **:*.* .                .    *  .:.     : . 

MG02980.1 	ASTFTSKGDQEGASQGTETG-AEPLNG---GRSFNSGKEDDTGASLSESPK---VFDPIE
NCU01961.1	---FTAKGSQ--PKQAFEAGNVEEWDGNAVEHKTNSTTYGENSTAMEDEGKGKTIFDPIQ
FG08889.1 	------------TPEAMLTE----------------RSPLRISQRRNEPDSSGRVFDPIE
AN4736.1  	------------SKAALEPE----------------KAPTPMTPVLSKEDAG--FIDPVA
          	            .  .  .                           ..   .  .:**: 

MG02980.1 	NKESWSKLLKKRIVPKC-EHDEPCISLQTKKPGINCG1RSFYICGRPLGPSGEKEKGTE~
NCU01961.1	NKVSWSKLLGKRVVPKC-EHGEDCQSLVTKKPGINCG1RSFYMCARPLGPSGNKETGTE~
FG08889.1 	AKESWSKLLGKRVAPRC-EHDEPCISLTTKKPGVNC-~--------------------V1
AN4736.1  	SKQDWSKLFTKKPPPKCGEHGEECISLKTKKPGPNFG~RTFWICPRPLGPSGNKEKGTE~
          	 * .****: *:  *:*.**.* * ** ***** * .  :   .  . ..:......:  

MG02980.1 	WRCGTFIWSSDWNASQQT
NCU01961.1	FRCKTFIWSSDWSGRQ--
FG08889.1 	LEKHVWIVRGD-------
AN4736.1  	WRCSTFIWANDWDSSTM-
          	 .  .:*  .* ..
```
